# The Intake of Energy and Selected Nutrients by Thai Urban Sedentary Workers: An Evaluation of Adherence to Dietary Recommendations

**DOI:** 10.1155/2014/145182

**Published:** 2014-12-02

**Authors:** Katiya Ivanovitch, Jeeranun Klaewkla, Rewadee Chongsuwat, Chukiat Viwatwongkasem, Wanicha Kitvorapat

**Affiliations:** ^1^Department of Nutrition, Faculty of Public Health, Mahidol University, 420/1 Ratchawithi Road, Ratchathewi, Bangkok 10400, Thailand; ^2^Department of Biostatistics, Faculty of Public Health, Mahidol University, 420/1 Ratchawithi Road, Ratchathewi, Bangkok 10400, Thailand; ^3^Regional Health Promotion Center 8, Nakhon Sawan, 157 Moo 1, Phahonyothin Road, Muang District, Nakhon Sawan 6000, Thailand

## Abstract

Rapid changes in Thailand's nutrition and lifestyles have led to increasing diet-related pathologies among people with sedentary occupations. This study examines the extent to which the dietary intake of nutrients and energy by a sample of Thai sedentary workers conforms to the Thai Dietary Reference Intakes (Thai DRIs). The nutrients and energy intake estimates were based on self-reported information collected with a single 24-hour dietary recall and nonweighed 2-day food record. The study participants were Thai adults aged 20–50 years employed in sedentary occupations. A convenience sample of 215 healthy individuals (75 males and 140 females) was based on four randomly selected worksites in the Bangkok metropolitan area. For male participants, the study found a median energy intake of 1,485 kcal/day, with 54.4% of energy coming from carbohydrate, 15.9% from protein, and 29.6% from fat. Females' median energy intake was 1,428 kcal/day, 56% of which came from carbohydrate, 16.2% from protein, and 28.6% from fat. Both genders showed insufficient intake of fiber and most micronutrients. This study provides the material for preventive public health interventions focusing on nutrition-related diseases affecting Thailand's rapidly growing sedentary workforce.

## 1. Introduction

The objective of this study is to examine nutrition-related risks of overweight/obesity for a group of sedentary workers in the Bangkok metropolitan area. The main methodological step in that process is a comparison of selected nutrients and energy intakes of a sample of 215 sedentary workers with the Thai Dietary Reference Intakes (Thai DRIs). Epidemiological studies typically use the term sedentary to describe physically inactive individuals [1]. A somewhat more nuanced view has also been adopted to describe sedentary behavior as a prolonged sitting rather than a severe lack of physical activity. Sedentary lifestyles are characterized by low energy expenditure levels, usually less than 1.5 METs (metabolic equivalents) [2]. Evidence also shows that sedentary behaviors are associated with growing health risks [3, 4].

A prolonged occupational sitting is a fact of daily life in both developed and developing economies. In Thailand, for example, automation, urbanization, and mass transit travel have led to increasingly sedentary lifestyles [5, 6]. As a result, 13 million people, or 33% of the active civilian labor force, are becoming seriously affected by overweight and obesity [7, 8]. The acute nature of that public health problem is highlighted by the fact that 47.3% of men and 57.5% of women in adult Thai population have been classified as overweight/obese [9], based on the Asian BMI criteria (BMI ≥ 23 kg/m^2^) [10]. This growing public health problem has negative implications for Thailand's productive capacity, because excessive body weight leads to increased mortality risks [11, 12] caused by type 2 diabetes, cardiovascular diseases, and various forms of cancer [13, 14]. In addition to their negative impact on productivity and workplace safety, these illnesses also require a broad range of medical treatments, creating an increasing burden on public health services and constant upward pressures on healthcare costs [15, 16].

As in many other countries, Thailand's public health authorities have used dietary guidelines to inform and assist consumers in making healthy nutrition choices and in following healthy lifestyles. However, no specific public health intervention has been conducted with regard to sedentary workers, partly because of the lack of adequate data on their nutrient and energy intakes. A research was, therefore, needed to examine the extent to which estimated energy and nutrients intakes of adults with sedentary occupations conform to the current Thai DRIs [17]. This paper is a first attempt to fill that important gap in public health research based on evidence collected in Thailand.

## 2. Materials and Methods

### 2.1. Workplaces and Participants

The data collection phase for this study commenced in February 2012 and was completed in April 2012. The study's research protocol was reviewed and approved by the Institutional Review Research in Human Subjects Committee, Faculty of Public Health, Mahidol University (COA. number: MUPH 2012-001). At each worksite, data were collected for two weeks. Letters were sent to each worksite to explain the study's purpose and objectives. Informed consent forms were read and signed by all study subjects. The participants in this study were working adults aged 20–50 years. They were part of a convenience sample based on four randomly selected worksites in the Bangkok metropolitan area. All the participants satisfied sedentary job characteristics: sitting occupation/desk-bound jobs, involving at least 4 hours per day of sitting during work hours. Most of the sedentary jobs were clerical, administrative, or data processing jobs. Excluded from the study were (1) people taking medication to control blood pressure or plasma lipids, (2) athletes/sports persons, (3) individuals on diets, (4) pregnant and/or lactating women, (5) people with self-reported chronic diseases, (6) persons with diseases that induce weight loss or weight gain, and (7) persons with physical or psychological disabilities.

The sample size of the study was determined by a priori power statistical analysis using the G^*^Power, Version 3.1.9.2, developed by the University of Düsseldorf, Germany [18]. Following this statistical procedure, a small-to-medium effect size of 0.4 [19], the *α*-error = 0.05 and the power = 0.8, gave a sample size of 66 participants for comparison of three male and female nutrition status groups. After a 15% adjustment to account for likely dropouts, a total sample size of 75 participants was obtained for each gender group. The final result was that a sample size of at least 150 (75 men and 75 women) study subjects would be required. For this study, 470 subjects from four worksites in the Bangkok metropolitan area were invited to participate. But since only 300 subjects (100 men and 200 women) agreed to participate, the response rate was 64%. The data collection was done by means of self-administered questionnaires handed out to study participants.

### 2.2. Anthropometric Measurements

The body weight (BW), body mass index (BMI), body fat percentage (%BF), and basal metabolic rate (BMR) were measured by Body Composition Analyzer (Tanita TBF 410, Tanita Corp., Tokyo, Japan) with the bioelectrical impedance (BIA) technology for accurate analysis. Waist circumference (WC) was also measured.

(1) WC was measured using a nonstretch, spring weighted anthropometric tape with a tension meter attached in the horizontal plane. The measure is taken midway between the inferior margin of the ribs and the superior border of the iliac crest at the end of normal expiration, as recommended by the World Health Organization and the International Diabetes Federation [20]. Abdominal measurements in this study were done in accordance with Asian-Pacific recommendations for WC cut-off points [21], where WC < 90 cm for males and WC < 80 cm for females are considered normal, while WC ≥ 90 cm for males and WC ≥ 80 cm for females indicate abdominal obesity.

(2) BW, BMI, and %BF were measured using the Tanita body composition digital scale. The Tanita scale used in this study had the “foot-to-foot” BIA technology. The bioelectrical impedance analysis (BIA) is performed with a nonharmful, low dose, conduction of electrical waves to measure weight and body composition. The Tanita scale was placed on a flat surface, and participants were asked to remove their shoes and heavy objects (such as jackets, wallets, and accessories) for measurement accuracy. Measurement protocols were given to participants one day before the tests. Measurement guidelines included (i) no meals and/or drinks three hours prior to measurements, (ii) no strenuous exercise 12 hours prior to measurements, (iii) no alcohol consumption 24 hours prior to measurements, (iv) absence of menstruation at the time of measurements, and (v) empty bladder before measurements. Participants were measured at approximately the same time of day. In case participants did not, or could not, observe the premeasurement guidelines, appointments were made to meet with them again when the guidelines were met. The body weight was measured in kilograms with one decimal point, waist circumference was determined to ±0.1 cm, and percent body fat was measured in percentage with ±0.1%. Asian criteria were used for BMI classifications [21] with the following cut-off points: BMI < 18.5 kg/m^2^ indicating underweight, BMI of 18.5–22.9 kg/m^2^ defined as a normal range, BMI of 23–24.9 kg/m^2^ considered as overweight, BMI of 25–30 kg/m^2^ defined as obesity class I, and BMI ≥ 30 kg/m^2^ defined as obesity class II. The threshold for overweight/obesity in this study was, therefore, defined as a BMI ≥ 23 kg/m^2^. Asian criteria for body fat percentage were also used to identify overweight/obesity with the following cut-off points: for males >20% for ages 20–29 and males >25% for ages 30–50; for females >24% for ages 20–29 and 35% for ages 30–50 [22].

### 2.3. Leisure-Time Physical Activity (LPTA) Assessment

The LPTA was self-reported using a modified version of Bouchard's [23] physical activity questionnaire. The questionnaire consisted of 13 open-ended daily physical activities. Duration of each activity was divided into 15 minutes per session, with the frequency of that activity per week. The energy expenditure was calculated according to the intensity of physical activities (kcal/kg/15 min). For each 15-minute period, energy expenditure is qualified on a scale from 1 to 9. Approximate median energy expenditure for each of the nine categories in kcal/kg per 15 minutes is applied to compute daily energy expenditure for each individual [23]. Briefly, subjects were asked to report all leisure physical activities that were performed at least 3 times per week in 15-minute sessions (frequency/week < 3 times, 3–5 times, and >5 times and time/session < 15 min, 15–30 min, 31–45 min, 46–60 min, and >60 min). Detailed information was then collected about the type of LTPA (such as walking, cycling, swimming, jogging, and gardening), as well as the frequency and duration of each LTPA reported.

### 2.4. Dietary Assessment

The dietary assessment for energy and nutrients intake was determined by a single 24-hour dietary recall and a nonweighed 2-day food record. The 24-hour dietary recall was conducted by interviewers who were trained and experienced nutritionists. An information session was organized to give each study participant instructions on how to record the daily food intake. In the food diaries, days were divided into seven eating occasions, namely, breakfast, lunch, dinner, and snacks (before breakfast, midmorning, afternoon, and late-evening snacks). Information on the type (including brand names) and amounts of food consumed was collected through an open entry format. For the verification and estimation of the size of individual food portions, the participants were instructed with the help of three-dimensional food models and household utensils to enhance the accuracy of portion size estimation. Participants were also given oral instructions on how to record their food intake and were shown how to use the household scale. Written instructions were incorporated in the food diaries as well, along with contact information in case any questions arose during the recording of food consumption. Participants were asked to record all food and beverages consumed for two consecutive days (Friday and Saturday) and to indicate dates and times when meals were taken. Since most of the meals reported as “brunch” were the subjects' first eating occasion of the day, breakfast and brunch were collapsed into one group referred to as breakfast. The importance of maintaining regular diets and recording all food and drink consumed was emphasized. The quality control of all food diaries was handled and reviewed by an experienced observer to avoid inconsistency and to maintain accurate data entries. After completion, the diaries were processed to record food quantities by experienced nutritionists using a standard manual on food portions and household measures [24]. The food codes were those used in the Thai Dietary Database (fourth edition), Institute of Nutrition, Mahidol University [25]. The calculation of nutrients was done by means of INMUCAL-Nutrient Software Version 2. While most macronutrients (carbohydrate, protein, and fat) could be found in that software's database, it was not possible to get all micronutrients for local food items. Consequently, micronutrients whose data were available in 80% or more of (except about 50% for zinc) food items in the INMUCAL database were included in the statistical analysis. The data on regional foods not covered by the software were sourced from the Thai Food Composition Table of the Nutrition Division, Department of Health, Ministry of Public Health of Thailand [26]. Adequacy of each nutrient intake was determined based on the 2003 Dietary Reference Intake for Thai People [17].

Incomplete food records and records for less than three days were excluded (76), leaving 224 complete records for data analysis (78 for men and 146 for women). The number of valid 24-hour dietary recalls and nonweighed 2-day food records were obtained for 78% and 73% of male and female participants, respectively. Nine nonplausible reporters, identified as subjects reporting energy intakes of <800 or >4,200 kcal/day for males or <600 and >3,500 kcal/day for females, and unrealistic values of other nutrients were excluded from the analysis [27]. The standardization of the exclusion procedure was guaranteed by the fact that the evaluation of the food diaries was done by the same nutritionist with a long-standing experience in the field of nutrition and epidemiological research. The valid data obtained for nutrients and energy intake was then compared with the Thai DRIs for adults aged 19–50 years. In this study, the Thai DRIs were based on the Recommended Dietary Allowances (RDA) for energy and most nutrients and on recommendations of Adequate Intake (AI) values for calcium and manganese. The Thai DRIs did not provide the tolerable upper intake level (UL), except for vitamin C, calcium, and sodium, as shown in Table 1 [17].

Another recommendation set by the Thai DRIs is the following daily macronutrient energy distribution for adults aged 19–50 years: 45%–65% from carbohydrate, 10%–15% from protein, and 20%–35% from fat.

Two variables—adherent and nonadherent—were used to measure participants' adherence to the Thai DRIs. Adherence to recommendations was defined as the level of energy and nutrient intake falling within 80–120% of the Thai DRIs. Nonadherence referred to insufficient or excessive intakes. Since the estimated average requirements (EAR) have not been established for the Thai DRIs, insufficient or inadequate energy and nutrients intake for each participant by age and sex was defined as that less than 80% of the Thai DRIs, estimated according to the formula RDA = 1.2 EAR of the US Institute of Medicine (IOM) [28]. Excessive intakes are those higher than the UL (for vitamin C, calcium, and sodium only) and those above 120% of the Thai DRIs.

### 2.5. Other Assessments

Smoking, socioeconomic factors, alcohol consumption, and medical history were also assessed by using a self-administered questionnaire. Based on the questionnaire, the participants were classified as those who never smoked in their life, former smokers (have not smoked in the past 12 months), and current smokers. For alcohol consumption, the participants were categorized into lifetime abstainers, former drinkers (have not drunk in the past 12 months), and current alcohol users. If participants reported that they were either current smokers or current alcohol users, they were asked to specify the frequency (i.e., daily or occasionally) of smoking or alcohol consumption.

### 2.6. Statistical Analysis

Predictive Analytics Software for Windows (PASW) Version 18 was used for data analysis. Descriptive statistics in this study are means and standard deviations for normally distributed continuous data and percentages of participant's adherence, or nonadherence, to dietary recommendations. Tests for normality were performed using the Kolmogorov-Smirnov test. Since not all the dietary intakes were distributed normally, median values and interquartile range (IQR) of the amounts estimated in a single 24-hour dietary recall and a nonweighed 2-day food record are also presented. A nonparametric test—Mann-Whitney *U* test—was used to identify median differences of macronutrient and energy intakes between genders and between nutritional status groups among males. The Kruskal Wallis test was used to identify differences of macronutrient and energy intakes between nutritional status groups among female participants. The Kruskal Wallis test was also used to identify the differences of BMI and energy and macronutrient intakes between four groups of eating occasions: 3 meals, 4 meals, 5 meals, and ≥6 meals. The Bonferroni corrections for multiple comparisons were then analyzed by the Mann-Whitney U test for group comparisons at the *P* value < 0.0125. The differences of the proportion of participants adhering to dietary recommendations among genders and nutritional status groups were also identified by using the chi-square test at *P* value < 0.05. Comparisons of the three-day average macronutrient and energy intakes between breakfast, lunch, and dinner were analyzed by using a nonparametric one-way repeated-measures ANOVA (Friedman's test) with the *P* value of <0.05 as a threshold for significance. That analysis was followed by comparing the average of macronutrient and energy intakes for breakfast, lunch, and dinner using nonparametric paired *t*-tests (Wilcoxon signed-rank tests). A Bonferroni correction was applied for the* post hoc* detection of significant pairwise differences, with the significance level set at *P* value < 0.0125.

## 3. Results

### 3.1. Sociodemographic and Anthropometric Profile

Table 2 presents the sociodemographic and anthropometric characteristics of study subjects consisting of 65.1% males and 34.9% females. The average age was 36.7 years (SD 8.1). Almost one-half of male participants were in a group older than 40 years. Among female participants, 38.6% were in the 30–39 age group and 39.3% were older than 40 years. More than a half of participants were single: 53.3% for males and 53.6% for females. The majority of study subjects (86.0%) had a bachelor degree or higher. Monthly incomes of less than 20,000 Thai baht were reported by 40% of females and by 26% of males. All participants were nonhabitual smokers and drinkers. Only 18.7% (*n* = 14) of males reported occasional smoking, while more than two-thirds of males (68.0%) and nearly half of females (45.7%) reported occasional drinking.

Based on BMI measurements, the prevalence of overweight/obesity among males was almost two times higher than among females: 64.0% and 35.7%, respectively. The prevalence of abdominal obesity was almost the same for male and female participants: 36.0% and 34.3%, respectively. One-half of males (50.7%) and one-fourth (25.7%) of females were in a high range of percent body fat. The sitting time during the working hours of all participants was between 4 and 9 hours, with an average of 6.0 hours per day (SD 1.1). The average of the leisure time energy expenditure was 124.9 kcal/day (SD 119.8), and the average basal metabolic rates were 1,552 kcal (SD 143) for men and 1,206 kcal (SD 111) for women.

### 3.2. Daily Macronutrient and Energy Intakes

A preliminary analysis using nonparametric Kruskal Wallis test identified the differences of energy and macronutrient intakes between age groups and sociodemographic characteristics at a significance level of *P* value < 0.05. The results showed that there were no significant differences in daily energy and nutrient intakes among the three age groups (20–29, 30–39, and ≥40) and other sociodemographic characteristics (education level, marital status, and monthly income) of participants. Consequently, in further analysis, all age groups and sociodemographic characteristics were combined, and the results are presented in Table 3.

The median values of energy and macronutrient intakes (carbohydrate, protein, and fat) showed significant differences between genders, with intakes by men being higher than those by women (*P* value < 0.05). The median energy intake for men was 1,485 kcal (IQR 270) and was 1,428 kcal (IQR 280) for women. That accounted for 71.2% of the Thai DRIs for men and for 81.1% for women; the difference was statistically significant at the *P* value < 0.05. The median carbohydrate intake was 203 grams/day (IQR 50) for men and 194 grams/day (IQR 43) for women, representing 54.4% and 56.0% of their total energy intake, respectively. The intakes of protein for men and women were 61 grams/day (IQR 15) and 56 grams/day (IQR 13), respectively, accounting for 15.9% of daily energy intake for men and 16.2% for women. Both genders showed higher consumption of protein compared to the Thai DRIs: 106.7% for men and 108.6% for women. The median daily intake of fat was 52 grams/day (IQR 18) for men and 44 grams/day (IQR 16) for women, representing 29.6% and 28.6% of their daily energy intake, respectively.

A comparison of the energy and macronutrient intake between nutritional status groups, classified by BMI (Table 4 for men and Table 5 for women), found that there was no significant difference in median daily energy and macronutrient intakes between normal weight and overweight/obese males and between underweight, normal weight, and overweight/obese females. The only exception was the carbohydrate intake by males. The median carbohydrate intake of overweight/obese males was 217 grams/day (IQR 50) and was 197 grams/day (IQR 50) for those of normal weight, with a statistically significant difference at *P* < 0.05.

### 3.3. Micronutrients Intake and Dietary Fiber

The results reported in Table 3 show that intakes of most micronutrients—calcium, potassium, vitamin A, vitamin E, vitamin B_1_, vitamin C, magnesium, selenium, and zinc—were less than 80% of the Thai DRIs. The intakes of phosphorus, vitamin B_2_ and niacin were found adequate in the range of 84.8%–110.6% of the Thai DRIs for both genders. The iron intake by males was adequate at 94.6% of the Thai DRIs, but it was very low—39.2% of the Thai DRIs—in the case of female participants, indicating a statistically significant difference of iron consumption between genders (*P* value < 0.05). A significant difference between genders was also found with regard to the intake of niacin, magnesium, and zinc. The sodium intake exceeded by far the Thai DRIs: males' median intake of 1,967 mg/day (IQR 973) was 135.3% of DRIs, and the females' intake of 2,021 mg/day (IQR 1,091) was 168.4% of DRIs. The median intake of dietary fiber of 7.8 grams/day (IQR 4.8) for men and 8.0 grams/day (IQR 4.8) for women accounted for 31.2% and 32.0% of the Thai DRIs, respectively. Results reported in Tables 4 and 5 show that there were no statistically significant differences for the median intake of micronutrients and fiber between nutritional status groups of both genders.

### 3.4. Adherence to Dietary Recommendations

A proportion of study participants conforming to the Thai DRIs for energy and nutrient intakes are shown in Table 3. Less than a half of female study subjects (48.6%) and less than one-fourth of males (21.3%) adhered to energy recommendations, showing a statistically significant difference between genders (*P* value < 0.05). That showed that more than three-quarters of male participants (78.7%) and half of females (49.3%) had an insufficient energy intake. With regard to the protein intake, 76.0% of men and 65.7% of women adhered to the Thai DRIs.

In the area of micronutrients, 62.7% of men and 65.0% of women adhered to phosphorus intake recommendations. But the adherence to the Thai DRIs was much lower for calcium: only 24.0% of men and 18.6% of women in our study adhered to calcium intake recommendations. There was also a very low, 5.0%, adherence to iron intake by women. By contrast, 42.7% of men were found to be in compliance with iron intake recommendations. About three-quarters of males (72.0%) and two-thirds (67.9%) of females adhered to sodium intake recommendations. For vitamin C and niacin intakes, the rates of adherence ranged from 49.3% to 73.3% for both genders. However, the adherence to recommended vitamin A intakes was very low: 10.7% for males and 11.4% for females. A lack of adherence to the Thai DRIs was also noted in the case of both genders with respect to intakes of fiber and other micronutrients, such as potassium, vitamin E, magnesium, selenium, and zinc.

There were no significant differences in adherence to recommended energy and nutrient intakes between nutritional status groups. The only significant difference (*P* value < 0.05) was found with regard to females' vitamin C intakes. The highest level—61.9%—of adherence to the recommended vitamin C intake was shown in the group of underweight females, followed by 55.1% in a normal weight group and by 36.0% in overweight/obese female participants.

### 3.5. Meal Patterns

Table 6 shows that significant differences (*P* value < 0.05) were found with respect to energy and macronutrient intakes for the three daily meals and snacks. Male participants showed the highest energy intake in kcal/day for dinner (517; IQR 183), followed by lunch (501; IQR 129), breakfast (392; IQR 126), and snacks (75; IQR 195). There were no statistically significant differences of males' energy intake (kcal/day) and the intakes of carbohydrate, protein, and fat (grams/day) between lunch and dinner. The highest energy intake for women was at lunch (461; IQR 138), followed by dinner (432; IQR 162), breakfast (372; IQR 142), and snacks (217; IQR 179). The highest carbohydrate intake (grams/day) by females was found at lunch, followed by dinner, breakfast, and snacks. The females' intakes of protein and fat were not statistically different between lunch and dinner.

**Figure 1:**
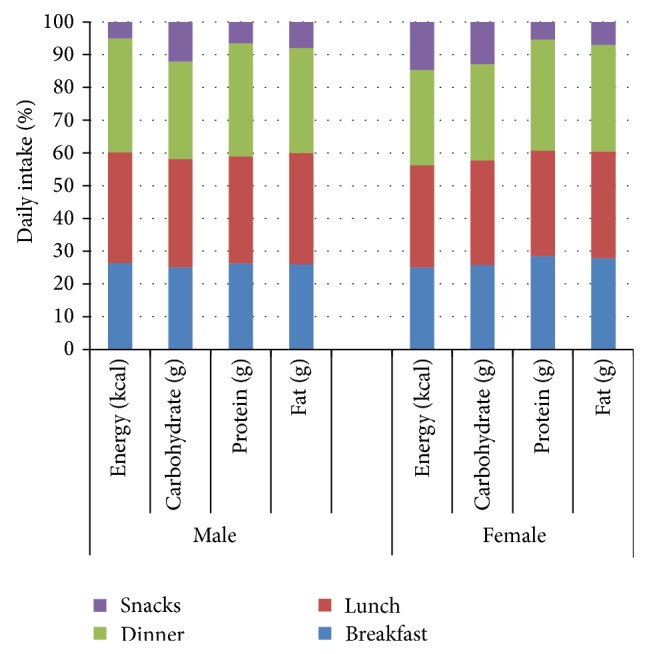
Daily percentage of energy distribution for breakfast, lunch, dinner, and snacks.

The daily energy distribution derived from the three meals and snacks is presented in Figure 1.

**Figure 2:**
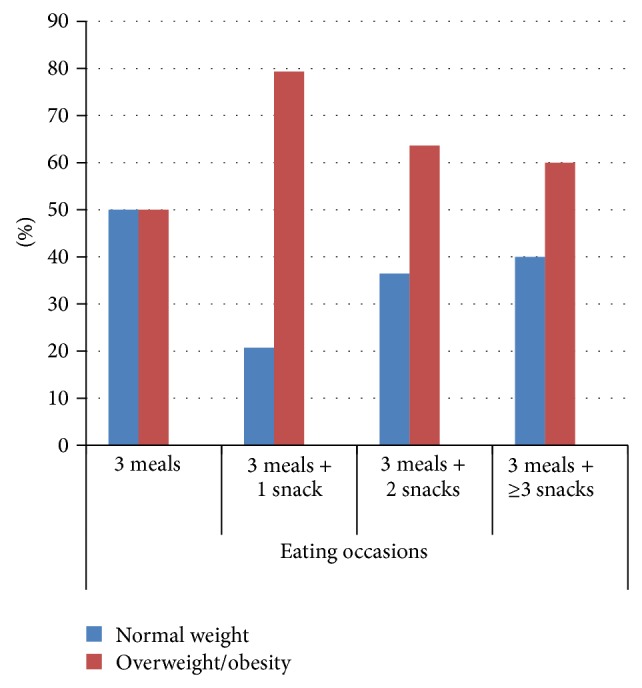
Comparison of eating occasions and nutritional status of male participants.

**Figure 3:**
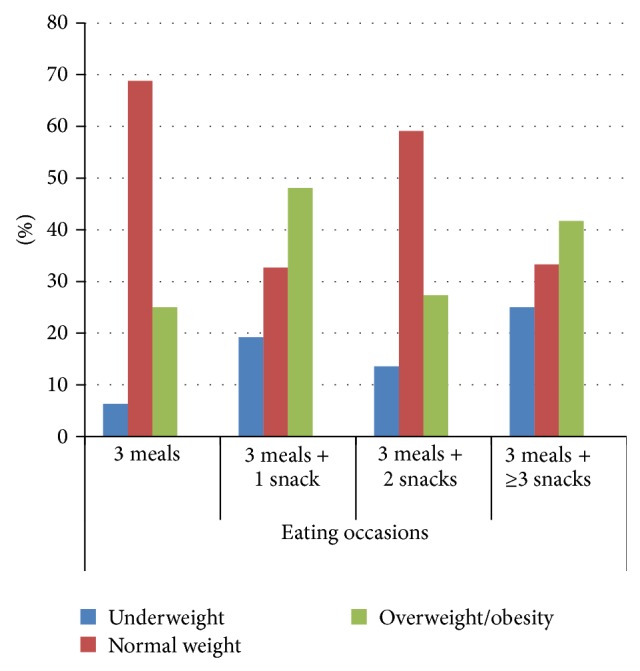
Comparison of eating occasions and nutritional status of female participants.

Table 7 shows the comparison of BMI and macronutrient intakes for different eating occasions. That comparison showed no statistically significant difference between eating occasions for both genders. Participants of both genders with eating occasions ≥6 times/day also reported the highest level of energy and all macronutrient intakes. Female participants' energy intake of 1,488 kcal/day for eating occasions ≥6 times/day was significantly higher than 1,343 kcal/day for those who had eating occasions 3 times/day (*P* value < 0.0125). For male participants, the highest carbohydrate intake—237 grams/day—was reported by those who had eating occasions 5 times/day, and that was significantly higher than 195 grams/day for those who had only 3 eating occasions per day (*P* value < 0.0125). Among females, the highest carbohydrate intake—209 grams/day—was recorded for those with eating occasions ≥6 times/day, followed by 205 grams/day for those with 5 eating occasions/day and 179 grams/day for those who had 3 eating occasions/day (*P* value < 0.05). Figures 2 and 3 further illustrate that subjects were more frequently classified as normal weight if they had 3 eating occasions/day relative to those who had 4, 5, or 6 eating occasions/day.

Normal weight and overweight/obese male participants showed no difference with regard to the energy intake from the three daily meals and snacks.

There were no statistically significant differences of protein and fat intake among eating occasions for both genders.

## 4. Discussion

Thailand is a developing country where rapid socioeconomic changes are affecting health and lifestyles of urban dwellers with sedentary occupations. By directly influencing the people's nutrition patterns and physical activity, these changes are creating serious health risks for the growing population subgroup of sedentary workers. While a large amount of research has been devoted to health risks facing people in sedentary occupations, a relatively limited attention was paid to problems of their nutrition patterns and dietary intakes.

This study shows the seriousness of diet-related health risks for Thai sedentary workers because it found that 64.0% of males and 35.7% of females in that population subgroup were found to be overweight/obese (BMI ≥ 23 kg/m^2^). Also, 50.7% of males and 25.7% of females in sedentary occupations were in the high range of the percent body fat, and more than one-third (34.9%) of the entire study sample had abdominal obesity. These findings are similar to those reported for other countries where research focused on health hazards of occupational sitting. The prominent examples of such studies are Choi et al. [29] in the United States, Mummery et al. [30] in Australia, Larsson et al. [31] in Sweden, Ishizaki et al. [32] in Japan, and Shimokawa et al. [33] in China.

The study by Mummery et al. [30] about the relationship between sedentary work and overweight/obesity in Australia reports that the probability of having a BMI ≥ 25 kg/m^2^ is greatly increased when the occupational sitting time is ≥5 hours/day, even in cases where physical activity levels reflect national guidelines. Our study found that the average sitting time in the Thai sample of sedentary workers was 6 hours/day, suggesting that this particular population subgroup in Thailand is exposed to a much higher risk of overweight/obesity and attendant diet-related pathologies.

That risk is also increased by our findings that the sedentary workers in our sample fell far short of adhering to the Thai DRIs for most nutrients, such as dietary fiber, potassium, calcium, iron, vitamin A, vitamin E, vitamin B_1_, vitamin B_2_, vitamin C, magnesium, selenium, and zinc. There were only few nutrients, mainly from the macronutrient category, where the study subjects conformed to the Thai DRIs.

The medians of energy intakes in our sample were also lower than those recommended in the Thai DRIs. The reported energy intakes of 1,485 kcal/day for males and 1,428 kcal/day for females accounted for only 71.2% and 80.1%, respectively, of the recommended Thai DRIs [17]. Relatively low energy intakes for both genders have also been reported in the Thai NHES IV whose estimates were based on a 24-hour dietary recall in a subsample of 2,969 people aged 15 years and over [34]. That study reported energy intakes of 1,600 kcal/day for males and 1,300 kcal/day for females. This may be partly due to the common problem that people tend to underreport their dietary intakes in 24-hour dietary recalls and in food records [35]. More generally, underreporting of energy intake is a well-known problem in self-reported dietary assessments and has been well documented in studies conducted in both developed [36, 37] and developing countries [38]. Using the Goldberg cut-off 1 test [39], we found that male and female participants in our sample underreported their energy intake by 26% and 11%, respectively. (The ratio of mean energy intake (EI) and the basal metabolic rate (BMR) < 1.35 is considered to represent underreporting. The average basal metabolic rate in this study was 1,552 kcal/day for men and 1.201 kcal/day for women. Thus, the energy requirement (BMR × 1.35) for male and female sedentary workers would be 2,000 kcal/day and 1,600 kcal/day, resp.) These results show that males' underreporting of energy intake is twice as high as the females' underreporting in a sample where 64% of males and 35.7% of females were classified as overweight/obese. Our findings are similar to other studies showing that overweight/obese individuals tend to underreport their dietary intake by as much as 20–50% [40, 41].

Median macronutrient intakes of carbohydrates and fats found in this study were all within their respective Thai DRIs [17]. The carbohydrate intake accounted for 54.4% of the total daily energy for men and for 56.0% for women. These results are almost identical to those reported in the latest national dietary survey by NHES IV of the general Thai adult population, showing that the percentage of energy distribution from carbohydrates was 52.7% for men and 55.5% for women [34, 42]. It is interesting to note that earlier Thai nutrition surveys found that the carbohydrate intake as a percentage of total energy declined from 78.9% in 1960 [43] to 62.1% in 2006 [44]. That was considered to result from a shift away from the traditional Thai staples that were high in carbohydrates to modern nutrition patterns mainly based on protein-rich food groups.

Protein intakes found in this study were higher than those recommended by the Thai DRIs. That higher protein intake was consistent with the research showing that, over the last five decades, the Thai protein consumption as part of the daily energy intake increased from 10% in 1960 [43] to 16% in 2009 [34]. It is interesting to note, however, that although the amount of protein intake in this study was higher than the Thai DRIs, it was still consistent with the Thai NHES IV, where recommended protein intakes were defined by acceptable macronutrient distribution ranges (AMDRs) developed by the US Institute of Medicine (IOM) [45].

With respect to the fat intake, this study found that the consumption of fats accounted for 29.6% of male and 28.6% of female daily energy. Our findings also conform to the data reported in Thai nutrition surveys, indicating that the fat consumption increased from 8.9% of total energy intake in 1960 to 28% in 2009 [34, 43, 46]. This is another confirmation of how much the traditional Thai diets, based on starchy staples, have been replaced by diets higher in animal protein [47]. These dietary changes are directly linked to the rising prevalence of dyslipidemia in the Thai population [48].

Micronutrients, required for virtually all metabolic and developmental processes, have been found with inadequate intakes in our study sample. Lower-than-recommended intakes were noted in the case of potassium, calcium, iron, vitamin A, vitamin E, vitamin B_1_, vitamin B_2_, magnesium, selenium, and zinc, indicating that, similar to other developing countries, micronutrient deficiencies are still a major public health problem in Thailand [49]. Inadequate micronutrient intakes are considered to reflect changing nutrition patterns associated with increasing risks of overweight, obesity, and other diet-related diseases [43].

Calcium intakes for most participants (80%) in our study were insufficient and amounted to 60% of the Thai DRIs. The NHES IV survey also reported very low calcium intakes. In that survey, the calcium intake for Thai adults (19–59 years age group) represented only 30% of the Thai DRIs, and only 24.1% of them consumed milk and dairy products [34]. Historically, milk has never been an important part of Thai nutrition because there were virtually no dairy cattle until the second half of the last century [50]. The inadequate calcium intake is an additional health risk for an already vulnerable sedentary population, because cross-sectional and longitudinal studies have widely reported that calcium deficiencies had adverse effects on blood pressure, metabolic balance, and weight management [51–53]. Female study participants showed an insufficient intake of iron. At 9.5 mg/day, the females' iron intake in our study is only 39.2% of the Thai DRIs. That is confirming the finding in the latest Thai dietary survey, which reported that, at 8.5 mg/day, the iron dietary intake by adult Thai women accounted for about one-third of the Thai DRIs [34]. Although it seems that the situation has improved somewhat in recent years, the iron deficiency had been noted as an important issue for the Thai female population since the mid-1970s [54].

Our finding of insufficient intakes of antioxidant vitamins (vitamins A, C, and E), which are present in many plant-based foods, is an indication of Thailand's low consumption of fruits and vegetables. That is confirmed by the Thai NHES III and IV nutrition surveys [55]. These surveys showed that only 36.5% of participants consumed fruit and 68.0% consumed vegetables on a daily basis in relatively small portions of 100 grams to 162.5 grams [34]. Antioxidant deficiencies compromise the oxidative state of the individual and could, therefore, increase the risk of comorbidities associated with overweight/obesity, such as heart disease, certain types of cancer, and insulin resistance [56, 57]. The low intake of selenium and zinc may be typical of our sample, drawn from the Bangkok-based sedentary workers, but the insufficient consumption of fish and shellfish is not typical of the diet followed by the general Thai population [44].

Our study indicates that the Thai's sodium intake remains excessive. Almost one-third of our study subjects reported a sodium consumption ≥2,400 mg/day, confirming similar results of the high intake of sodium in many studies, including the NHES IV [58, 59]. Typically, Thai dishes use condiments such as fish sauce, soya sauce, oyster sauce, table salt, and shrimp paste that are very high in sodium content. Excessive sodium intake is a worldwide problem [60]. Thailand, like many other countries, continues to conduct public health campaigns to lower the sodium consumption [61], because some evidence is showing that a high sodium intake may be contributing to problems of hypertension, cardiovascular diseases, and chronic kidney ailments in the Thai population [62].

We also found an insufficient potassium intake by both genders. Potassium is a very important mineral to regulate blood pressure and lower the risks of stroke and heart disease. Since potassium is mainly found in plant-based foods, its deficiency in the Thai diet is reflecting the already mentioned low consumption of fruits and vegetables.

Significantly inadequate fiber consumption is another important problem found in this study. Fiber intakes of 7.8 grams/day for men and 8.0 grams/day for women are far below the 25 grams/day recommended by the Thai DRIs. Our findings are consistent with those of the Thai NHES IV, reporting fiber intakes of 7.5 grams/day for males and 8.5 grams/day for females [55]. The problem of the low dietary fiber intake in this study is due to inadequate consumption of fruits, vegetables, and grains, partly because these foods are not readily available in the office area or in office canteens. Most of the study subjects reported having breakfast at the office, consisting mainly of coffee, pastries, sandwiches, congee (based on rice with small quantities of minced pork/chicken), or grilled pork and sticky rice. Only a few study subjects reported having a complete meal (composed of carbohydrate, protein, fruits, and vegetables) or a salad for breakfast. Our interviewers observed that lunch was typically taken in office canteens where not enough vegetables were served. Changes toward greater consumption of fiber-rich foods are an important public health issue in Thailand, because that would lower the risk of metabolic syndrome, diabetes, and cardiovascular diseases [63, 64].

Our study also shows that progress has to be made toward improving the quality of food in worksite canteens, as has been the case in some other countries [65, 66].

The study's findings about meal pattern characteristics (Figure 1) show that breakfast provides 26% of daily energy for both genders, which is only slightly above the 20%–25% general range for meals energy distribution. Lunch provided 34% of daily energy for men and 32% for women (well within the general range of 30%–35%). The dinner supplied 35% of daily energy for men and 30% for women. Snacks provided 5% of daily energy for men and 12% for women (the recommended range is 10%–20%). These results show that our study participants had a proper energy distribution between breakfast, lunch, dinner, and snacks and that most of their daily energy came from main meals.

Our results presented in Table 7 confirm those of previous studies that a higher number of eating occasions lead to higher energy intake and that higher carbohydrate consumption was associated with higher BMI measurements. A recent study reported that more daily meals were consumed by obese than by lean women [67]. Snacks, in particular, were identified to contain a higher proportion of carbohydrates and a smaller proportion of fats than regular meals [68]. The growing trend of grazing rather than following the traditional pattern of three proper meals is considered to be a major factor in the etiology of obesity. Forslund et al. showed that obese Swedish women snacked more often and had higher energy intake than their lean peers [67]. The French women with a habit of frequent snacking were also found to have a higher energy intake than those who did not snack [69].

### 4.1. Strengths and Limitations of This Study

The main limitation of this study resides in underreporting of energy intake. That is a well-known and well-documented problem of self-reported dietary assessment methods such as the 24-hour dietary recall and a nonweighed food record. We have calculated, for example, that male and female participants in this study underreported their respective energy intakes by 26% and 11%. Similar problems have also been noted in national nutrition surveys, including those conducted in Thailand [70, 71]. The 24-hour dietary recall relies on the respondents' memory, and the food record has a heavy record-keeping burden, showing, in some cases, that energy intakes decrease with increasing days of reporting the food consumption [72].

The other limitation of this study is due to the limitation of the Thai dietary database (INMUCAL-NV.2.0). That problem is discussed by Satheannoppakao et al. [59]. For example, some commonly used Thai food ingredients such as fermented fish (“Nam-Pla-Ra”) are high in sodium, but the nutrient analysis shows no results for sodium content. Also, this software version cannot calculate the proportion of saturated fat in total fat. This nutrient analysis can provide up to 34 items of information on energy and nutrients for 2,054 food products with 50%–99.8% food biochemical references. But for vitamin B_6_ and vitamin B_12_, the program gives only 6.5% and 9.1% of biochemical references, which made it impossible for us to report these vitamins' intakes.

The important strength of this study is in methods and procedures used during the data collection and processing. Precise and comprehensive instructions were given to study subjects for the completion of a single 24-hour dietary recall and a nonweighed 2-day food record during orientation sessions. Food models were also used to enhance the accuracy of (a) portion estimates, (b) the record of food items consumed, and (c) the daily variation in dietary intakes.

In spite of a relatively small sample size (*n* = 215), this study's results on energy and nutrient intakes are quite similar and in some cases identical to those produced by studies, such as the Thai National Health Examination Surveys (NHES IV 2008-2009 [34]), based on much larger samples using the same dietary assessment methods. That is most probably because the relevant sample in that study was of a similar size to the one we used. Indeed, the NHES survey was based on a subsample of 2,969 people aged 1–60 years, but only 935 people were in the 15–59 age group. If, according to the physical activity assessment in that survey, these 935 subjects in that age group are further classified into three groups—nonsedentary, sedentary, and unemployed—the resulting sample size would very likely be similar to the sample size (*n* = 215) used in our study. Moreover, our study included only healthy sedentary workers having no diet-related diseases, but the NHES IV subsample did not exclude unhealthy subjects who might have had diet-related pathologies.

These study sample comparisons lead us to believe that our dietary assessment is suitable and that it can be representative of the nutrition patterns of Thailand's urban sedentary workers.

### 4.2. Implications of the Study

Our research shows that energy and nutrient intakes of Thai sedentary workers should be brought in greater compliance with the Thai DRIs. The way to do that is by improving public health messages about healthy dietary guidelines. That means that there is a need for public health intervention initiatives to inform and educate the public about healthy eating and healthy lifestyles. That is the new line of research highlighted by our study. Another possible topic for further study is the improvement of food quality in worksite eateries. A much broader study may also be warranted, based on a considerably larger sample size than the one we used, to evaluate diet-related health risks of the sedentary population.

## 5. Conclusions

This study examined the extent to which energy and nutrient intakes of a sample of sedentary workers in the Bangkok metropolitan area conform to the Thai DRIs. A declining trend noted for energy and carbohydrate intakes and an increasing consumption of protein and fat are reflecting shifts in the Thai diet away from traditional carbohydrate-rich staples to nutrition patterns based on proteins and fats. Intakes of dietary fiber and most micronutrients, including calcium, vitamins A, C, and E, magnesium, selenium, and zinc, did not meet the Thai DRIs. Higher intakes of foods rich in fat and lower intakes of fiber and antioxidants are putting sedentary workers at a greater risk of diet-related chronic diseases. Our study suggests that there is a need to improve dietary patterns for sedentary workers through worksite wellness and nutrition education initiatives. Based on our research, these nutrition education programs should emphasize that increasing consumption of certain nutrients found in this study (e.g., fat and sodium) and lower intakes of others (e.g., fiber, calcium, vitamin A, vitamin E, and vitamin C) are linked to a greater risk of cardiovascular diseases and certain forms of cancers. Worksites offer a large and captive audience to promote dietary behavior that would increase people's adherence to the recommended energy and nutrient intakes and physical activity programs that would reduce health risks inherent in sedentary lifestyles. Improving the quality of foods served in worksite canteens by providing healthier meal selections should be a priority in Thailand's efforts to promote public health.

This first and unique attempt to focus on nutrition problems of people in sedentary occupations is a positive contribution to Thailand's public health research. By offering a thorough evaluation of the extent to which sedentary workers' nutrition patterns conform to the Thai DRIs, this study's findings can be used in nutrition intervention programs to prevent diet-related diseases in this high-risk population subgroup.

## Figures and Tables

**Table 1:** The Thai Dietary Reference Intakes (Recommended Dietary Allowances and Adequate Intakes of energy and selected nutrients) for adults aged 19–50 years.

Energy and nutrients	Sex				Tolerable upper intake levels
	Males		Females	
	Aged 19–30 yrs	Aged 31–50 yrs	Aged 19–30 yrs	Aged 31–50 yrs
(1) Energy (kcal/d)	2,150	2,100	1,750	1,750	ND
(2) Protein (g/d)	57	57	52	52	ND
(3) Fiber (g/d)	25	25	25	25	ND
(4) Calcium (mg/d)	800	800	800	800	2,500
(5) Phosphorus (mg/d)	700	700	700	700	ND
(6) Sodium (mg/d)	500–1,475	475–1,450	400–1,200	400–1,200	2,400
(7) Potassium (mg/d)	2,450–4,100	2,450–4,100	2,050–3,400	2,050–3,400	ND
(8) Iron (mg/d)	10.4	10.4	24.7	24.7	ND
(9) Vitamin A (*µ*g/d)	700	700	600	600	ND
(10) Vitamin E (mg/d)	15	15	15	15	ND
(11) Vitamin B_1_ (mg/d)	1.2	1.2	1.1	1.1	ND
(12) Vitamin B_2_ (mg/d)	1.3	1.3	1.1	1.1	ND
(13) Niacin (mg/d)	16	16	14	14	ND
(14) Vitamin C (mg/d)	90	90	75	75	2,000
(15) Magnesium (mg/d)	310	320	250	260	ND
(16) Selenium (*µ*g/d)	55	55	55	55	ND
(17) Zinc (mg/d)	13	13	7	7	ND

ND = not determined due to lack of suitable data.

Source: Thai Dietary Reference Intakes for Energy and Selected Nutrients (2003). The report may be accessed via http://nutrition.anamai.moph.go.th/temp/main/view.php?group=2&id=132.

**Table 2:** Sociodemographic and anthropometric profile of participants.

Characteristics	All (*n* = 215)	Men (*n* = 75)	Women (*n* = 140 )
	Number (percentage)	Number (percentage)	Number (percentage)
(1) Age in years			
20–29	49 (22.8)	18 (24.0)	31 (22.1)
30–39	75 (34.9)	21 (28.0)	54 (38.6)
≥40	91 (42.3)	36 (48.0)	55 (39.3)
(2) Marital status			
Single	114 (53.3)	40 (53.3)	75 (53.6)
Married	93 (43.4)	34 (45.3)	59 (42.1)
Divorced/widowed/separated	7 (3.3)	1 (1.4)	6 (4.3)
(3) Educational level			
Diploma	30 (14.0)	14 (18.7)	16 (11.4)
Bachelor degree	122 (56.7)	43 (57.3)	79 (56.4)
Master degree or higher	63 (29.3)	18 (24.0)	45 (32.2)
(4) Monthly income (baht)			
Less than 20,000	77 (35.8)	20 (26.7)	57 (40.7)
20,000–29,999	61 (28.4)	23 (30.7)	38 (27.1)
30,000–39,000	33 (15.3)	8 (10.6)	25 (17.9)
≥40,000	44 (20.5)	24 (32.0)	20 (14.3)
(5) Smoking			
Never smokers	194 (90.2)	55 (73.3)	139 (99.3)
Former smokers	7 (3.3)	6 (8.0)	1 (0.7)
Current smokers	14 (6.5)	14 (18.7)	0 (0.0)
Occasional smokers	14 (100.0)	14 (100.0)	0 (0.0)
(6) Alcohol users			
Lifetime abstainers	60 (27.9)	13 (17.3)	47 (33.6)
Former drinkers	40 (18.6)	11 (14.7)	29 (20.7)
Current alcohol users	115 (53.5)	51 (68.0)	64 (45.7)
Occasional drinkers	115 (100.0)	51 (100.0)	64 (100.0)
(7) Nutritional status classification			
Body mass index (BMI)			
Underweight	21 (9.8)	0 (0.0)	21 (15.0)
Normal weight	96 (44.6)	27 (36.0)	69 (49.3)
Overweight/obese^a^	98 (45.6)	48 (64.0)	50 (35.7)
Percent body fat (%BF)			
Normal range	104 (74.3)	37 (49.3)	104 (74.3)
High range^b^	36 (25.7)	38 (50.7)	36 (25.7)
Waist circumference (WC)			
Normal waist	140 (65.1)	48 (64.0)	92 (65.7)
Abdominal obesity^c^	75 (34.9)	27 (36.0)	48 (34.3)
(8) Sitting time at work (hour/day)			
Mean ± SD	6.0 ± 1.1	5.7 ± 1.1	6.1 ± 1.1
(9) Leisure time energy expenditure (kcal/day)			
Mean ± SD	124.9 ± 119.8	157.4 ± 138.6	107.5 ± 104.8
(10) Basal metabolic rate (BMR) (kcal)			
Mean ± SD	1,323.8 ± 207.2	1,552.1 ± 143.0	1,201.5 ± 111.5
(11) Total energy intake: basal metabolic rate (EI: BMR)			
Mean ± SD	1.1 ± 0.2	1.0 ± 0.2	1.2 ± 0.2

^a^Overweight/obese: BMI ≥ 23 kg/m^2^ (Asian definition).

^b^High range: males of ages 20–29 > 20%, ages 30–50 > 25%; females of ages 20–29 > 24%, ages 30–50 > 35%.

^c^Abdominal obesity: males ≥ 90 cm; females ≥ 80 cm (Asian definition).

**Table 3:** Energy and nutrient intakes classified by genders.

Variables	Male (*n* = 75)					Female (*n* = 140)				
	Median (IQR)	%DRI	Adherent *n* (%)	Nonadherent; *n* (%)		Median (IQR)	%DRI	Adherent *n* (%)	Nonadherent; *n* (%)	
				Insufficient *n* (%)	Excessive *n* (%)				Insufficient *n* (%)	Excessive *n* (%)
Energy (kcal)	**1,485 (270)** ^ a^	**71.2** ^ b^	**16 (21.3)** ^ c^	59 (78.7)	0 (0.0)	**1,428 (280)** ^ a^	**80.1** ^ b^	**68 (48.6)** ^ c^	69 (49.3)	3 (2.1)
Carbohydrate (g)	**203 (50)** ^ a^	NA	NA	NA	NA	**194 (43)** ^ a^	NA	NA	NA	NA
Protein (g)	**61 (15)** ^ a^	106.7	57 (76.0)	0 (0.0)	18 (24.0)	**56 (13)** ^ a^	108.6	92 (65.7)	9 (6.4)	39 (27.9)
Animal protein (g)	30 (14)^a^	NA	NA	NA	NA	31 (11)^a^	NA	NA	NA	NA
Fat (g)	**52 (18)** ^ a^	NA	NA	NA	NA	**44 (16)** ^ a^	NA	NA	NA	NA
Carbohydrate (energy percentage)	**54.4 (7.9)** ^ a^	NA	67 (89.3)	5 (6.7)	3 (4.0)	**56.0 (9.1)** ^ a^	NA	133 (95.0)	4 (2.9)	3 (2.1)
Protein (energy percentage)	15.9 (3.2)	NA	57 (76.0)	0 (0.0)	18 (24.0)	16.2 (3.0)	NA	92 (65.7)	9 (6.4)	39 (27.9)
Fat (energy percentage)	29.6 (6.6)	NA	60 (80.0)	3 (4.0)	12 (16.0)	28.6 (7.6)	NA	118 (84.3)	5 (3.6)	17 (12.1)
Dietary fiber (g)	7.8 (4.8)	31.2	0 (0.0)	75 (100.0)	0 (0.0)	8 (4.8)	32.0	1 (0.7)	139 (99.3)	0 (0.0)
Calcium (mg)	492 (200)	62.5	18 (24.0)	57 (76.0)	0 (0.0)	444 (199)	55.2	26 (18.6)	114 (81.4)	0 (0.0)
Phosphorus (mg)	684 (200)	97.7	47 (62.7)	18 (24.0)	10 (13.3)	655 (147)	93.6	91 (65.0)	31 (21.1)	18 (12.9)
Sodium (mg)	1,967 (973)	**135.3** ^ b^	54 (72.0)	0 (0.0)	21 (28.0)	2,021 (1,091)	**168.4** ^ b^	95 (67.9)	0 (0.0)	45 (32.1)
Potassium (mg)	1,251 (549)	51.1	0 (0.0)	75 (100.0)	0 (0.0)	1,219 (501)	59.5	4 (2.9)	136 (97.1)	0 (0.0)
Iron (mg)	9.8 (5.1)	**94.6** ^ b^	**25 (33.3)** ^ c^	32 (42.7)	18 (24.0)	9.5 (3.3)	**39.2** ^ b^	**7 (5.0)** ^ c^	133 (95.0)	0 (0.0)
Vitamin A (*µ*g)	346 (567)	49.5	8 (10.7)	50 (66.7)	17 (22.7)	316 (409)	52.6	16 (11.4)	95 (67.9)	29 (20.7)
Vitamin E (mg)	0.04 (0.4)	0.2	0 (0.0)	75 (100.0)	0 (0.0)	0.17 (0.4)	1.1	3 (2.1)	134 (95.8)	3 (2.1)
Vitamin B_1_ (mg)	0.9 (0.5)	**74.3** ^ b^	24 (32.0)	45 (60.0)	6 (8.0)	0.9 (0.4)	**79.7** ^ b^	49 (35.0)	71 (50.7)	20 (14.3)
Vitamin B_2_ (mg)	1.3 (.8)	**96.9** ^ b^	26 (34.7)	26 (34.7)	23 (30.6)	1.2 (0.6)	**110.6** ^ b^	55 (39.3)	28 (20.0)	57 (40.7)
Niacin (mg)	**13.8 (4.5)** ^ a^	86.3	55 (73.3)	16 (21.3)	4 (5.3)	**11.9 (4.1)** ^ a^	84.8	97 (69.3)	36 (25.7)	7 (5.0)
Vitamin C (mg)	64 (103)	71.4	41 (54.7)	34 (45.3)	0 (0.0)	68 (122)	90.5	69 (49.3)	71 (50.7)	0 (0.0)
Magnesium (mg)	**15.8 (19.7)** ^ a^	**6.7** ^ b^	0 (0.0)	75 (100.0)	0 (0.0)	**22.9 (26.4)** ^ a^	**10.3** ^ b^	0 (0.0)	140 (100.0)	0 (0.0)
Selenium (*µ*g)	0.3 (1.1)	0.7	0 (0.0)	75 (100.0)	0 (0.0)	0.4 (0.7)	0.7	0 (0.0)	140 (100.0)	0 (0.0)
Zinc (mg)	**2.1 (1.0)** ^ a^	**16.1** ^ b^	0 (0.0)	75 (100.0)	0 (0.0)	**2.3 (1.2)** ^ a^	**33.5** ^ b^	0 (0.0)	140 (100.0)	0 (0.0)

^a^ *P* values are for differences in median dietary intake between genders (Mann-Whitney *U* test); significant differences shown in bold at *P* value < 0.05.

^b^ *P* values are for differences in %DRIs between genders (Mann-Whitney *U* test); significant differences shown in bold at *P* value < 0.05.

^c^ *P* values are for differences in proportion of participants' adherence to recommendations between genders (chi-square test); significant differences shown in bold at *P* value < 0.05.

**Table 4:** Energy and nutrient intakes of male participants classified by nutritional status.

Variables	BMI < 23 kg/m^2^ (n = 27)					BMI ≥ 23 kg/m^2^ (n = 48)				
	Median (IQR)	%DRI	Adherent n (%)	Nonadherent; n (%)		Median (IQR)	%DRI	Adherent n (%)	Nonadherent; n (%)	
				Insufficient n (%)	Excessive n (%)				Insufficient n (%)	Excessive n (%)
Energy (kcal)	1,496 (306)	71.2	12 (44.4)	15 (55.6)	0 (0.0)	1,536 (306)	71.5	22 (45.8)	26 (54.2)	0 (0.0)
Carbohydrate (g)	**197 (50)** ^ a^	NA	NA	NA	NA	**217 (50)** ^ a^	NA	NA	NA	NA
Protein (g)	60 (17)	106.1	21 (77.8)	0 (0.0)	6 (22.2)	61 (17)	107.8	36 (75.0)	0 (0.0)	12 (25.0)
Animal protein (g)	29.6 (12.9)	NA	NA	NA	NA	29.4 (11.6)	NA	NA	NA	NA
Fat (g)	52 (15)	NA	NA	NA	NA	51 (15)	NA	NA	NA	NA
Carbohydrate (energy percentage)	52.3 (8.6)	NA	22 (81.5)	3 (11.1)	2 (7.4)	55.0 (7.9)	NA	45 (93.7)	1 (2.1)	2 (4.2)
Protein (energy percentage)	16.4 (4.1)	NA	11 (40.7)	0 (0.0)	16 (59.3)	15.8 (3.2)	NA	18 (37.5)	0 (0.0)	30 (62.5)
Fat (energy percentage)	30.3 (8.7)	NA	21 (77.8)	1 (3.7)	5 (18.5)	29.2 (6.3)	NA	39 (81.2)	2 (4.2)	7 (14.6)
Dietary fiber (g)	6.9 (3.3)	27.6	0 (0.0)	27 (100.0)	0 (0.0)	8.6 (4.4)	34.4	0 (0.0)	48 (100.0)	0 (0.0)
Calcium (mg)	498.6 (226.4)	62.3	6 (22.2)	21 (77.8)	0 (0.0)	492.3 (302.8)	60.9	12 (25.0)	36 (75.0)	0 (0.0)
Phosphorus (mg)	615.2 (224.2)	87.9	13 (48.1)	10 (37.0)	4 (14.8)	707.8 (179.5)	101.1	34 (70.8)	8 (16.7)	6 (12.5)
Sodium (mg)	2,159 (908)	141.4	19 (70.4)	0 (0.0)	8 (29.6)	1,842 (1,117)	129.5	35 (72.9)	0 (0.0)	13 (27.1)
Potassium (mg)	1,150 (543)	46.9	0 (0.0)	27 (100.0)	0 (0.0)	1,264 (521)	51.6	0 (0.0)	48 (100.0)	0 (0.0)
Iron (mg)	8.7 (5.5)	85.9	9 (33.3)	12 (44.4)	6 (22.2)	10.3 (4.6)	97.4	23 (49.9)	13 (27.1)	12 (25.0)
Vitamin A (*µ*g)	346 (445.2)	50.1	4 (14.8)	18 (66.7)	5 (18.5)	316 (712.4)	47.9	4 (8.3)	32 (66.7)	12 (25.0)
Vitamin E (mg)	0.04 (0.3)	0.05	0 (0.0)	27 (100.0)	0 (0.0)	0.06 (0.4)	0.32	0 (0.0)	48 (100.0)	0 (0.0)
Vitamin B_1_ (mg)	0.8 (0.5)	67.2	9 (33.3)	18 (66.7)	0 (0.0)	0.9 (0.6)	76.1	15 (31.2)	27 (56.3)	6 (12.5)
Vitamin B_2_ (mg)	1.2 (0.9)	93.7	9 (33.3)	9 (33.3)	9 (33.3)	1.3 (0.8)	96.9	17 (35.4)	17 (35.4)	14 (29.2)
Niacin (mg)	12.9 (4.2)	81.3	12 (44.4)	13 (48.2)	2 (7.4)	14.2 (4.7)	87.7	29 (60.4)	17 (35.4)	2 (4.2)
Vitamin C (mg)	59.6 (111.9)	66.2	12 (44.4)	15 (55.6)	0 (0.0)	65.0 (98.8)	71.8	22 (45.8)	26 (54.2)	0 (0.0)
Magnesium (mg)	13.9 (12.1)	4.5	0 (0.0)	27 (100.0)	0 (0.0)	21.7 (23.5)	6.7	0 (0.0)	48 (100.0)	0 (0.0)
Selenium (*µ*g)	0.2 (1.3)	0.5	0 (0.0)	27 (100.0)	0 (0.0)	0.4 (0.9)	0.7	0 (0.0)	48 (100.0)	0 (0.0)
Zinc (mg)	1.8 (0.9)	14.3	0 (0.0)	27 (100.0)	0 (0.0)	2.2 (1.1)	16.9	0 (0.0)	48 (100.0)	0 (0.0)

^a^ *P* values are for differences in median dietary intakes between nutritional status groups (Mann-Whitney *U* test); significant differences shown in bold at *P* value < 0.05.

**Table 5:** Energy and nutrient intakes of female participants classified by BMI.

Variables	BMI < 18.5 kg/m^2^ (*n* = 21)					BMI 18.5–22.9 kg/m^2^ (*n* = 69)					BMI ≥ 23 kg/m^2^ (*n* = 50)				
	Median (IQR)	%DRI	Adherent; *n* (%)	Nonadherent; *n* (%)		Median (IQR)	%DRI	Adherent; *n* (%)	Nonadherent; *n* (%)		Median (IQR)	%DRI	Adherent; *n* (%)	Nonadherent; *n* (%)	
				Insufficient *n* (%)	Excessive *n* (%)				Insufficient *n* (%)	Excessive *n* (%)				Insufficient *n* (%)	Excessive *n* (%)
Energy (kcal)	1,334 (263)	76.3	8 (38.1)	13 (61.9)	0 (0.0)	1,441 (300)	82.3	38 (55.1)	30 (43.5)	1 (1.4)	1,401 (289)	79.4	22 (44.0)	26 (52.0)	2 (4.0)
Carbohydrate (g)	178.5 (39.5)	NA	NA	NA	NA	202.8 (43.6)	NA	NA	NA	NA	192 (42)	NA	NA	NA	NA
Protein (g)	54.8 (8.6)	105.3	16 (76.2)	1 (4.8)	4 (19.0)	56.7 (13.4)	109.0	45 (65.2)	4 (5.8)	20 (29.0)	58 (15)	109.8	31 (62.0)	4 (8.0)	15 (30.0)
Animal protein (g)	30.9 (8.4)	NA	NA	NA	NA	32.5 (12.2)	NA	NA	NA	NA	29.1 (10.5)	NA	NA	NA	NA
Fat (g)	47.4 (15.7)	NA	NA	NA	NA	43.8 (15.8)	NA	NA	NA	NA	43.9 (15.6)	NA	NA	NA	NA
Carbohydrate (energy percentage)	52.2 (7.7)	NA	19 (90.4)	1 (4.8)	1 (4.8)	56.9 (7.8)	NA	64 (92.8)	3 (4.3)	2 (2.9)	53.5 (8.7)	NA	50 (100.0)	0 (0.0)	0 (0.0)
Protein (energy percentage)	16.7 (2.4)	NA	5 (23.8)	0 (0.0)	16 (76.2)	15.9 (2.6)	NA	24 (34.8)	2 (2.9)	43 (62.3)	16.4 (3.0)	NA	13 (26.0)	1 (2.0)	36 (72.0)
Fat (energy percentage)	30.9 (7.3)	NA	17 (81.0)	2 (9.5)	2 (9.5)	26.9 (7.4)	NA	59 (85.5)	2 (2.9)	8 (11.6)	29.3 (7.9)	NA	42 (84.0)	1 (2.0)	7 (14.0)
Dietary fiber (g)	8.1 (3.6)	32.4	0 (0.0)	21 (100.0)	0 (0.0)	9.3 (5.1)	37.2	1 (1.4)	68 (98.6)	0 (0.0)	8.2 (5.1)	32.8	0 (0.0)	50 (100.0)	0 (0.0)
Calcium (mg)	490.8 (234.7)	61.3	3 (14.3)	18 (85.7)	0 (0.0)	445.0 (210.8)	55.3	16 (23.2)	53 (76.8)	0 (0.0)	427.3 (180.3)	52.1	7 (14.0)	43 (86.0)	0 (0.0)
Phosphorus (mg)	657.4 (151.8)	93.9	13 (61.9)	6 (28.6)	2 (9.5)	660.3 (148.9)	94.3	45 (65.2)	14 (20.3)	10 (14.5)	647.9 (142.4)	92.6	33 (66.0)	11 (22.0)	6 (12.0)
Sodium (mg)	1,902 (926)	158.5	16 (76.2)	0 (0.0)	5 (23.8)	1,979 (1,068)	164.9	51 (73.9)	0 (0.0)	18 (26.1)	1,979 (1,068)	183.1	28 (56.0)	0 (0.0)	22 (44.0)
Potassium (mg)	1,236 (322)	60.3	1 (4.8)	20 (95.2)	0 (0.0)	1,229 (473)	59.9	2 (2.9)	67 (97.1)	0 (0.0)	1,196 (646)	58.3	1 (2.0)	49 (98.0)	0 (0.0)
Iron (mg)	9.2 (2.4)	37.4	0 (0.0)	21 (100.0)	0 (0.0)	9.8 (3.7)	40.3	3 (4.3)	66 (95.7)	0 (0.0)	9.3 (3.9)	38.9	4 (8.0)	46 (92.0)	0 (0.0)
Vitamin A (*µ*g)	310.5 (350.9)	51.8	2 (9.5)	15 (71.4)	4 (19.0)	323.3 (402.5)	51.2	11 (15.9)	43 (62.3)	15 (21.7)	311.5 (355.8)	51.9	3 (6.0)	37 (74.0)	10 (20.0)
Vitamin E (mg)	0.2 (0.7)	6.6	1 (4.8)	20 (95.2)	0 (0.0)	0.2 (0.4)	8.8	1 (1.4)	66 (95.7)	2 (2.9)	0.1 (0.4)	9.6	1 (2.0)	48 (96.0)	1 (2.0)
Vitamin B_1_ (mg)	0.8 (0.4)	72.3	5 (23.8)	13 (61.9)	3 (14.3)	0.9 (0.4)	79.8	27 (39.1)	35 (50.7)	7 (10.1)	0.9 (0.4)	82.2	17 (34.0)	23 (46.0)	10 (20.0)
Vitamin B_2_ (mg)	1.0 (0.5)	92.3	10 (47.6)	6 (28.6)	5 (23.8)	1.3 (0.8)	117.0	23 (33.3)	13 (18.8)	33 (47.8)	1.2 (0.5)	111.5	22 (44.0)	9 (18.0)	19 (38.0)
Niacin (mg)	11.8 (3.4)	84.6	13 (61.9)	8 (38.1)	0 (0.0)	11.9 (3.9)	85.2	38 (55.1)	26 (37.7)	5 (7.2)	11.4 (4.1)	78.8	22 (44.0)	26 (52.0)	2 (4.0)
Vitamin C (mg)	86.1 (106.5)	114.8	**13 (61.9**)^a^	8 (38.1)	0 (0.0)	77.5 (138.5)	103.4	**38 (55.1)** ^a^	31 (49.9)	0 (0.0)	50.8 (70.3)	67.8	**18 (36.0)** ^a^	32 (64.00)	0 (0.0)
Magnesium (mg)	15.5 (28.5)	10.1	0 (0.0)	21 (100.0)	0 (0.0)	22.8 (20.8)	10.1	0 (0.0)	69 (100.0)	0 (0.0)	24.4 (31.1)	10.8	0 (0.0)	50 (100.0)	0 (0.0)
Selenium (*µ*g)	0.4 (0.7)	0.7	0 (0.0)	21 (100.0)	0 (0.0)	0.4 (1.0)	0.7	0 (0.0)	69 (100.0)	0 (0.0)	0.4 (0.7)	0.7	0 (0.0)	50 (100.0)	0 (0.0)
Zinc (mg)	2.7 (1.4)	37.9	0 (0.0)	21 (100.0)	0 (0.0)	2.4 (1.2)	33.8	0 (0.0)	69 (100.0)	0 (0.0)	2.2 (1.2)	31.6	0 (0.0)	50 (100.0)	0 (0.0)

^a^ *P* values are for differences in proportion of participants' adherence to recommendations between nutritional status groups (chi-square test); significant differences shown in bold at *P* value < 0.05.

**Table 6:** Differences of macronutrient and energy intakes for 3 main meals (breakfast, lunch, and dinner).

	Breakfast	Lunch	Dinner	Snacks
	Median (IQR)	Median (IQR)	Median (IQR)	Median (IQR)
Male				
Energy (kcal)	392 (126)^a,b,c,d^	501 (129)^**a**,**b**,**f**^	517 (183)^a,**c**,**g**^	75 (195)^**a**,**d**,**f**,**g**^
Carbohydrate (g)	54 (20)^**a**,**b**,**c**,**d**^	71 (21)^**a**,**b**,**f**^	64 (27)^a,**c**,**g**^	26 (28)^**a**,**d**,**f**,**g**^
Protein (g)	16 (7)^a,**b**,**c**,**d**^	20 (6)^**a**,**b**,**f**^	21 (8)^a,**c**,**g**^	4 (5)^**a**,**d**,**f**,**g**^
Fat (g)	13 (7)^a,**b**,**c**,**d**^	17 (7)^**a**,**b**,**f**^	16 (10)^a,**c**,**g**^	4 (6)^**a**,**d**,**f**,**g**^
Female				
Energy (g)	372 (142)^**a**,**b**,**c**,**d**^	461 (138)^**a**,**b**,**e**,**f**^	432 (162)^a,**c**,**e**,**g**^	217 (179)^**a**,**d**,**f**,**g**^
Carbohydrate (g)	50 (18)^**a**,**b**,**c**,**d**^	62 (20)^**a**,**b**,**e**,**f**^	57 (22)^**a**,**c**,**e**,**g**^	25 (34)^**a**,**d**,**f**,**g**^
Protein (g)	16 (8)^a,**b**,**c**,**d**^	18 (7)^**a**,**b**,**f**^	19 (8)^a,**c**,**g**^	3 (4)^**a**,**d**,**f**,**g**^
Fat (g)	12 (8)^a,**b**,**c**,**d**^	14 (8)^**a**,**b**,**f**^	14 (9)^a,**c**,**g**^	3 (5)^**a**,**d**,**f**,**g**^

^a^ *P* values are for differences among the three meals and snacks (Friedman's test); significant differences shown in bold at *P* value < 0.05.

^**b**,**c**,**d**,**e**,**f**,**g**^Significantly different from breakfast at *P* value < 0.0125 (Wilcoxon nonparametric *t*-test with Bonferroni correction).

**Table 7:** Comparison of BMI and macronutrient intakes for different eating occasions.

	Number of eating occasions/day			
	3	4	5	≥6
Male	(*n* = 30)	(*n* = 29)	(*n* = 11)	(*n* = 5)
BMI (kg/m^2^)^*^	24.1 (3.8)	25.4 (3.3)	22.9 (2.2)	24.9 (4.6)
Energy (kcal/d)^**^	1,455 (22)^a^	1,578 (222)^a^	1,622 (344)^a^	1,684 (418)^a^
Carbohydrate (g/d)^**^	195 (49)^a,b,c^	218 (43)^a,b^	237 (60)^a,c^	215 (37)^a^
Protein (g/d)^**^	60 (13)	61 (14)	56 (13)	73 (24)
Fat (g/d)^**^	48 (13)	52 (17)	58 (23)	62 (25)
Female	(*n* = 32)	(*n* = 52)	(*n* = 44)	(*n* = 12)
BMI (kg/m^2^)^*^	21.7 (3.4)	22.8 (3.4)	21.5 (2.8)	22.6 (5.8)
Energy (kcal/d)^**^	1,343 (265)^a,d^	1,380 (266)^a^	1,460 (299)^a^	1,488 (149)^a,d^
Carbohydrate (g/d)^**^	179 (48)^a,c,d^	197 (36)^a^	205 (44)^a,c^	209 (54)^a,d^
Protein (g/d)^**^	56 (12)	56 (13)	56 (52)	60 (14)
Fat (g/d)^**^	44 (19)	44 (15)	44 (16)	50 (16)

^*^Mean (SD), ^**^median (IQR).

^a^Significant differences between 4 categories of eating occasions (Kruskal Wallis test), *P* value < 0.05.

^b,c,d^Significant differences between 2 categories of eating occasions (Mann-Whitney *U* test with Bonferroni correction), *P* value <0.0125.

## References

[1] Hamilton M. T., Hamilton D. G., Zderic T. W. (2007). Role of low energy expenditure and sitting in obesity, metabolic syndrome, type 2 diabetes, and cardiovascular disease. *Diabetes*.

[2] Pate R. R., O'Neill J. R., Lobelo F. (2008). The evolving definition of “sedentary”. *Exercise and Sport Sciences Reviews*.

[3] Bertrais S., Beyeme-Ondoua J. P., Czernichow S., Galan P., Hercberg S., Oppert J. M. (2005). Sedentary behaviors, physical activity, and metabolic syndrome in middle-aged French subjects. *Obesity Research*.

[4] Katzmarzyk P. T., Church T. S., Craig C. L., Bouchard C. (2009). Sitting time and mortality from all causes, cardiovascular disease, and cancer. *Medicine and Science in Sports and Exercise*.

[5] Popkin B. M. (1998). The nutrition transition and its health implications in lower-income countries. *Public Health Nutrition*.

[6] Popkin B. M. (1999). Urbanization, lifestyle changes and the nutrition transition. *World Development*.

[7] Aekplakorn W., Hogan M. C., Chongsuvivatwong V., Tatsanavivat P., Chariyalertsak S., Boonthum A., Tiptaradol S., Lim S. S. (2007). Trends in obesity and associations with education and Urban or rural residence in Thailand. *Obesity*.

[8] Aekplakorn W. (2009). *The Report of the 4th Thai National Health Examination Survey in 2008-2009*.

[9] Aekplakorn W., Inthawong R., Kessomboon P., Sangthong R., Chariyalertsak S., Putwatana P., Taneepanichskul S. (2014). Prevalence and trends of obesity and association with socioeconomic status in Thai adults: national health examination surveys, 1991–2009. *Journal of Obesity*.

[10] WHO Expert Consultation (1998). Appropriate body-mass index for Asian populations and its implications for policy and intervention strategies. *The Lancet*.

[11] Freedman D. M., Ron E., Ballard-Barbash R., Doody M. M., Linet M. S. (2006). Body mass index and all-cause mortality in a nationwide US cohort. *International Journal of Obesity*.

[12] Adams K. F., Schatzkin A., Harris T. B., Kipnis V., Mouw T., Ballard-Barbash R., Hollenbeck A., Leitzmann M. F. (2006). Overweight, obesity, and mortality in a large prospective cohort of persons 50 to 71 years old. *The New England Journal of Medicine*.

[13] Must A., Spadano J., Coakley E. H., Field A. E., Colditz G., Dietz W. H. (1999). The disease burden associated with overweight and obesity. *The Journal of the American Medical Association*.

[14] Malnick S. D. H., Knobler H. (2006). The medical complications of obesity. *QJM*.

[15] Raebel M. A., Malone D. C., Conner D. A., Xu S., Porter J. A., Lanty F. A. (2004). Health services use and health care costs of obese and nonobese individuals. *Archives of Internal Medicine*.

[16] Daviglus M. L., Liu K., Yan L. L., Pirzada A., Manheim L., Manning W., Garside D. B., Wang R., Dyer A. R., Greenland P., Stamler J. (2004). Relation of body mass index in young adulthood and middle age to medicare expenditures in older age. *Journal of the American Medical Association*.

[17] Nutrition Division (2003). *Dietary Referrence Intake for Thais 2003*.

[18] Faul F., Erdfelder E., Lang A.-G., Buchner A. (2007). G^*^Power 3: A flexible statistical power analysis program for the social, behavioral, and biomedical sciences. *Behavior Research Methods*.

[19] Cohen J. (1988). *Statistical Power Analysis for the Behavioral Sciences*.

[20] Wang J., Thornton J. C., Bari S. (2003). Comparisons of waist circumferences measured at 4 sites. *The American Journal of Clinical Nutrition*.

[21] World Health Organization Western Pacific Region, International Association for the Study of Obesity, International Obesity Task Force (2000). *The Asia Pacific Perspective: Redefining Obesity and Its Treatment*.

[22] Li L., Wang C., Bao Y., Peng L., Gu H., Jia W. (2012). Optimal body fat percentage cut-offs for obesity in Chinese adults. *Clinical and Experimental Pharmacology and Physiology*.

[23] Bouchard C., Tremblay A., Leblanc C., Lortie G., Savard R., Theriault G. (1983). A method to assess energy expenditure in children and adults. *The American Journal of Clinical Nutrition*.

[24] Sirichakwal P. P., Sranacharoenpong K., Tontisirin K. (2011). Food based dietary guidelines (FBDGs) development and promotion in Thailand. *Asia Pacific Journal of Clinical Nutrition*.

[25] Institute of Nutrition (2009). *Manual of INMUCAL-Nutrients*.

[26] Nutrition Division (2001). *Nutritive Values of Thai Foods*.

[27] Subar A. F., Thompson F. E., Kipnis V., Midthune D., Hurwitz P., McNutt S., McIntosh A., Rosenfeld S. (2001). Comparative validation of the block, willett, and national cancer institute food frequency questionnaires: the eating at America's Table Study. *The American Journal of Epidemiology*.

[28] Institute of Medicine (US) Food and Nutrition Board (1998). *Dietary Reference Intakes: A Risk Assessment Model for Establishing Upper Intake Levels for Nutrients*.

[29] Choi B., Schnall P. L., Yang H. (2010). Sedentary work, low physical job demand, and obesity in US workers. *American Journal of Industrial Medicine*.

[30] Mummery W. K., Schofield G. M., Steele R., Eakin E. G., Brown W. J. (2005). Occupational sitting time and overweight and obesity in Australian workers. *American Journal of Preventive Medicine*.

[31] Larsson C. A., Krøll L., Bennet L., Gullberg B., Råstam L., Lindblad U. (2012). Leisure time and occupational physical activity in relation to obesity and insulin resistance: a population-based study from the Skaraborg Project in Sweden. *Metabolism—Clinical and Experimental*.

[32] Ishizaki M., Morikawa Y., Nakagawa H., Honda R., Kawakami N., Haratani T., Kobayashi F., Araki S., Yamada Y. (2004). The influence of work characteristics on body mass index and waist to hip ratio in Japanese employees. *Industrial Health*.

[33] Shimokawa S., Chang H.-H., Pinstrup-Andersen P. (2009). Understanding the differences in obesity among working adults between Taiwan and China. *Asia Pacific Journal of Clinical Nutrition*.

[34] Aekplakorn W., Kessomboon P., Sangthong R., Chariyalertsak S., Putwatana P., Inthawong R., Nitiyanant W., Taneepanichskul S. (2011). Urban and rural variation in clustering of metabolic syndrome components in the Thai population: results from the fourth National Health Examination Survey 2009. *BMC Public Health*.

[35] Johansson L., Solvoll K., Bjørneboe G.-E. A., Drevon C. A. (1998). Under- and overreporting of energy intake related to weight status and lifestyle in a nationwide sample. *The American Journal of Clinical Nutrition*.

[36] Briefel R. R., Sempos C. T., McDowell M. A., Chien S., Alaimo K. (1997). Dietary methods research in the third National health and nutrition examination survey: underreporting of energy intake. *The American Journal of Clinical Nutrition*.

[37] Ferrari P., Slimani N., Ciampi A., Trichopoulou A., Naska A., Lauria C., Veglia F., Bueno-de-Mesquita H. B., Ocké M. C., Brustad M., Braaten T., Tormo M. J., Amiano P., Mattisson I., Johansson G., Welch A., Davey G., Overvad K., Tjønneland A., Clavel-Chapelon F., Thiebaut A., Linseisen J., Boeing H., Hemon B., Riboli E. (2002). Evaluation of under- and overreporting of energy intake in the 24-hour diet recalls in the European Prospective Investigation into Cancer and Nutrition (EPIC). *Public Health Nutrition*.

[38] Scagliusi F. B., Ferriolli E., Lancha A. H. (2006). Underreporting of energy intake in developing nations. *Nutrition Reviews*.

[39] Goldberg G. R., Black A. E., Jebb S. A., Cole T. J., Murgatroyd P. R., Coward W. A., Prentice A. M. (1991). Critical evaluation of energy intake data using fundamental principles of energy physiology: 1. Derivation of cut-off limits to identify under-recording. *European Journal of Clinical Nutrition*.

[40] Lichtman S. W., Pisarska K., Berman E. R. (1992). Discrepancy between self-reported and actual caloric intake and exercise in obese subjects. *The New England Journal of Medicine*.

[41] Goris A. H., Westerterp-Plantenga M. S., Westerterp K. R. (2000). Undereating and underrecording of habitual food intake in obese men: selective underreporting of fat intake. *American Journal of Clinical Nutrition*.

[42] (2003). *The Fifth National Nutirtional Survey of Thailand 2003*.

[43] Kosulwat V. (2002). The nutrition and health transition in Thailand. *Public Health Nutrition*.

[44] National Bureau of Argricultural Commodity and Food Standard (2006). *Food Consumption Data of Thailand*.

[45] Institute of Medicine (2011). *Dietary Reference Intakes for Energy, Carbohydrate, Fiber, Fat, Fatty Acids, Cholesterol, Protein, and Amino Acids 2005*.

[46] Jitnarin N., Kosulwat V., Rojroongwasinkul N., Boonpraderm A., Haddock C. K., Poston W. S. C. (2010). Risk factors for overweight and obesity among Thai adults: results of the national Thai food consumption survey. *Nutrients*.

[47] Pingali P. (2007). Westernization of Asian diets and the transformation of food systems: implications for research and policy. *Food Policy*.

[48] Aekplakorn W., Taneepanichskul S., Kessomboon P. (2014). Prevalence of dyslipidemia and management in the Thai population, National Health Examination Survey IV, 2009. *Journal of Lipids*.

[49] Díaz J. R., de las Cagigas A., Rodríguez R. (2003). Micronutrient deficiencies in developing and affluent countries. *European Journal of Clinical Nutrition*.

[50] Kehren T., Tisdell C. (1997). *The Thai Dairy Industry: Its Economic Evolution Raised by Land Rights and Cattle Diseases*.

[51] Torres M. R. S. G., Ferreira T. D. S., Carvalho D. C., Sanjuliani A. F. (2011). Dietary calcium intake and its relationship with adiposity and metabolic profile in hypertensive patients. *Nutrition*.

[52] Beydoun M. A., Gary T. L., Caballero B. H., Lawrence R. S., Cheskin L. J., Wang Y. (2008). Ethnic differences in dairy and related nutrient consumption among US adults and their association with obesity, central obesity, and the metabolic syndrome. *The American Journal of Clinical Nutrition*.

[53] Eilat-Adar S., Xu J., Loria C., Mattil C., Goldbourt U., Howard B. V., Resnick H. E. (2007). Dietary calcium is associated with body mass index and body fat in American Indians. *Journal of Nutrition*.

[54] Winichagoon P. (2002). Prevention and control of anemia: thailand experiences. *The Journal of Nutrition*.

[55] Satheannoppakao W., Aekplakorn W., Pradipasen M. (2009). Fruit and vegetable consumption and its recommended intake associated with sociodemographic factors: Thailand National Health Examination Survey III. *Public Health Nutrition*.

[56] Bonnefont-Rousselot D. (2004). The role of antioxidant micronutrients in the prevention of diabetic complications. *Treatments in Endocrinology*.

[57] Shah S., Iqbal M., Karam J., Salifu M., McFarlane S. I. (2007). Oxidative stress, glucose metabolism, and the prevention of type 2 diabetes: pathophysiological insights. *Antioxidants and Redox Signaling*.

[58] Pavadhgul P., Sunthonwaraluk S., Srisorachatr S., Temcharoen P. (2009). Dietary sodium intake by semi-quantitative food frequency questionnaire among undergraduate students of Mahidol University. *Journal of the Medical Association of Thailand*.

[59] Satheannoppakao W., Kasemsup R., Inthawong R. (2013). Sodium intake and socio-demographic determinants of the non-compliance with daily sodium intake recommendations: Thai NHES IV. *Journal of the Medical Association of Thailand*.

[60] World Health Organization (2007). *Report of a WHO Forum and Technical Meeting 5–7 October 2006, Paris*.

[61] Supornsilaphachai C. (2013). *Evolution of Salt Reduction Initiatives in Thailand: Lessons for Other Countries in the South-East Asia Region*.

[62] He F. J., MacGregor G. A. (2009). A comprehensive review on salt and health and current experience of worldwide salt reduction programmes. *Journal of Human Hypertension*.

[63] Galisteo M., Duarte J., Zarzuelo A. (2008). Effects of dietary fibers on disturbances clustered in the metabolic syndrome. *Journal of Nutritional Biochemistry*.

[64] Weickert M. O., Pfeiffer A. F. H. (2008). Metabolic effects of dietary fiber consumption and prevention of diabetes. *The Journal of Nutrition*.

[65] Lassen A., Thorsen A. V., Trolle E., Elsig M., Ovesen L. (2004). Successful strategies to increase the consumption of fruits and vegetables: results from the Danish “6 a day” work-site Canteen Model Study. *Public Health Nutrition*.

[66] Lassen A., Hansen K. S., Trolle E. (2007). Comparison of buffet and à la carte serving at worksite canteens on nutrient intake and fruit and vegetable consumption. *Public Health Nutrition*.

[67] Forslund H. B., Lindroos A. K., Sjöström L., Lissner L. (2002). Meal patterns and obesity in Swedish women—a simple instrument describing usual meal types, frequency and temporal distribution. *European Journal of Clinical Nutrition*.

[68] Bellisle F., Dalix A. M., Mennen L. (2003). Contribution of snacks and meals in the diet of French adults: a diet-diary study. *Physiology & Behavior*.

[69] Basdevant A., Craplet C., Guy-Grand B. (1993). Snacking patterns in obese French women. *Appetite*.

[70] Garriguet D. (2008). Under-reporting of energy intake in the Canadian Community Health Survey. *Health Reports/Statistics Canada, Canadian Centre for Health Information*.

[71] Kye S., Kwon S.-O., Lee S.-Y., Lee J., Kim B. H., Suh H.-J., Moon H.-K. (2014). Under-reporting of energy intake from 24-hour dietary recalls in the korean national health and nutrition examination survey. *Osong Public Health and Research Perspectives*.

[72] Liberato S. C., Bressan J., Hills A. P. (2009). Assessment of energy and macronutrient intake in young men: a comparison of 4-day food record and 24-hour dietary recall. *Revista de Nutrição*.

